# Factors related to patient adherence to the use of new oral anticoagulants*

**DOI:** 10.1590/1980-220X-REEUSP-2021-0191

**Published:** 2021-12-17

**Authors:** Paula Rocco Gomes Lima, Gisele Mara Silva Gonçalves, Roberta Cunha Matheus Rodrigues, Ana Railka de Souza Oliveira-Kumakura

**Affiliations:** 1 Universidade Estadual de Campinas, Faculdade de Enfermagem, Campinas, SP, Brazil.; 2 Pontifícia Universidade Católica de Campinas, Faculdade de Ciências Farmacêuticas, Campinas, SP, Brazil.

**Keywords:** Medication Adherence, Anticoagulants, Nursing, Cumplimiento de la Medicación, Anticoagulantes, Enfermería

## Abstract

**Objective:**

To characterize the self-reported adherence of patients with cardiovascular diseases to the use of new oral anticoagulants and to identify factors related to adherence to these drugs.

**Method:**

This is a descriptive, correlational, and cross-sectional study, carried out with outpatients. The collection of sociodemographic, clinical, and adherence data, through the Measurement of Adherence to Treatments, was made through telephone calls. Descriptive, correlation, and multiple linear regression analyses were used.

**Results:**

A total of 120 patients using new anticoagulants for 32.3 months, on average, participated in the study. More than half of the sample consisted of women, who were professionally inactive, with a mean age of 70.1 years and a mean family income of 6.7 minimum wages. The mean adherence score was 5.7, in a possible range between 1 and 6, indicating medication adherence. Inactive employment status, female sex, higher family income, and follow-up at a public outpatient clinic were related to greater adherence to these medications.

**Conclusion:**

The patients showed high adherence to new anticoagulants. Employment status, sex, family income, and type of outpatient follow-up were related to medication adherence, and should be considered in the design of interventions for this public.

## RESUMEN

**Objetivo:**

caracterizar la adhesión autorrelatada de los pacientes con enfermedades cardiovasculares al uso de nuevos anticoagulantes orales e identificar los factores relacionados a la adhesión a esos medicamentos.

**Método:**

estudio descriptivo, correlacional y transversal, con paciente ingresado. La recolección de datos sociodemográficos, clínicos y de adhesión, por medio de la Medida de Adhesión a los Tratamientos, ocurrió a través del teléfono. Se utilizaron el análisis descriptivo, correlacional y de regresión lineal múltiple.

**Resultados:**

participaron del estudio 120 pacientes que utilizaron nuevos anticoagulantes por 32,3 meses, aproximadamente. Más de la mitad de la muestra estuvo compuesta de mujeres jubiladas, con edad promedia de 70,1 años y remuneración familiar promedia de 6,7 sueldos mínimos. El score promedio de adhesión fue de 5,7, dentro de un período posible entre 1 y 6, lo que indicó adhesión farmacológica.

**Conclusión:**

los pacientes presentaron elevada adhesión a los nuevos anticoagulantes. Situación laboral, sexo, renta familiar y tipo de acompañamiento en ambulatorio estuvieron relacionados a la adhesión farmacológica, debiendo ser considerados en la delineación de intervenciones para ese público.

## INTRODUCTION

Medication adherence has increasingly been the focus of attention of health researchers, due to the strong impact that non-adherence to treatment can have on public and private health systems in Brazil and in the world. Among the consequences of non-adherence, the increase in the rate of hospitalizations and avoidables, and the direct and indirect costs of health care^(1–3)^ are highlighted. According to the World Health Organization (WHO) and researchers on the subject, adherence is defined as the degree of agreement between the health care provider’s recommendations and the patient’s behavior in relation to the proposed therapeutic regimen. Non-adherence, in its turn, has been considered by WHO as “a worldwide problem of impressive magnitudes” that has impacted the authorities^(2,4)^.

Therefore, as part of the health team working to mitigate the problem of non-adherence, based on well-structured evidence, nurses have a critical role in the process of optimizing patient adherence to medication, working in an educational manner in the stages of initiation and implementation of therapy to encourage persistence in the treatment, which leads to the prevention of health problems. However, even today, one of the great challenges for this professional is to promote and ensure the adherence of patients using polytherapies, considering that each drug has a dose, frequency, and specific times, which can be complex for the patient, contributing to non-adherence^(5)^ in the long term. In the case of patients being treated with oral anticoagulants (OAC), this reality is no different.

The OACs are drugs widely indicated for patients who have clinical conditions that undesirably activate blood clotting, acting both in the prevention and treatment of thromboembolic events^(6)^. For years, warfarin was the most used option in the treatment of diseases such as pulmonary and venous thromboembolism, atrial fibrillation (AF), acute myocardial infarction, heart valve prostheses, among others. However, over time, the limitations of this group of drugs became evident, as they are easily impacted by the action of various extrinsic and intrinsic factors, a fact that directly interferes with patient adherence to the drug^(7)^.

Given the recognition of this fact, new anticoagulants (NOAC) emerged with the proposal to ensure greater safety and the same therapeutic effects, with a reduction in adverse events^(8)^. However, even with the proposal of better results, international studies have shown that there is high fluctuation in the adherence rates of patients with different comorbidities to the use of NOAC, ranging between 23% and 90%^(9,10)^. Among patients with cardiovascular diseases (CVD), a population that has a high need for treatment and indication for the use of therapies with NOAC, adherence varies between 36.1% and 83.3%^(9)^.

The fluctuation of drug adherence rates can occur as a function of the method of adherence measurement, whether direct (direct observation of the patient using the drug, measurement of the rate of the active or metabolic principle in blood or urine, etc.) or indirect (use of self-report instruments, drug counts, etc.)^(11)^. Medication adherence can also be influenced by patient characteristics, duration of treatment, among others. Among the methods used, the indirect measurement through self-report instruments stands out, as it is one of the methods with lower cost, of easy application to assess medication adherence^(11)^.

Therefore, evaluating self-reported adherence and sociodemographic and clinical factors related to adherence in a sample of Brazilian patients with CVD is important, given the increase in the number of patients on NOAC, the complexity of treatment with NOAC, and the scarcity of studies aimed at analyzing adherence to these drugs on the national settings^(2–4)^.

Thus, this study aims to characterize the self-reported adherence of patients with cardiovascular diseases to the use of new oral anticoagulants and to identify the sociodemographic and clinical factors related to adherence to these medications.

## METHOD

### Design of Study

This is a descriptive, correlational, and cross-sectional study.

### Sample

The sample consisted of cardiovascular diseases patients on NOAC (dabigatran, rivaroxaban, or apixaban) in outpatient follow-up in the aforementioned fields of research, listed for convenience.

### Local

The study was carried out in a public university hospital and in a private cardiology clinic, both located in an inland city of the state of São Paulo.

### Selection Criteria

Patients over 18 years of age, using dabigatran, rivaroxaban, or apixaban for at least one month were included. Patients who presented communication deficits and who did not have a legal guardian to provide the information required by the research were excluded.

### Sample Definition

The sample size calculation for the purpose of evaluating the correlation between quantitative variables was based on the methodology of a sample calculation for a Pearson correlation coefficient. A test power of 90% was assumed, a significance level of 5%, an estimate for the correlation coefficient equal to 0.30, which according to Cohen (1992)^(12)^ is considered a coefficient of average degree, and a correlation coefficient equal to 0.00 is considered as the null hypothesis. The calculation resulted in a minimum sample of 112 participants. To perform sample calculation, the software G*Power 3.1.9.2 was used^(13)^.

### Data Collection

Data collection was carried out from October 2020 to January 2021 by the researcher and by three previously trained research assistants. A list of patients on NOAC and their telephone numbers were obtained in the aforementioned fields of research.

Patients and/or their legal guardians were approached and invited to participate in the study through telephone calls, due to restrictions imposed by the COVID-19 pandemic. After the presentation of the scope of the study and the verbalization of the free and informed consent, which was recorded at that time, data collection began. A sociodemographic and clinical characterization instrument previously developed for this research and the Brazilian version of the Measurement of Adherence to Treatment (MAT) scale were used^(14)^, which were applied through an interview, in a telephone call, with a loud, clear, and paused reading of the items, which were repeated as required by the participant. To complement the collection, the participants’ electronic medical records were consulted for more details on their clinical history and indication of treatment with NOAC.

The measurement of adherence to NOAC, made through the application of MAT, was considered the outcome variable, with the following variables being considered explanatory: sex (male or female), age (years), length of study (in years), education (illiterate, some primary education, complete primary education, some secondary education, complete secondary education, some higher education or complete higher education), color (white or nonwhite – black, brown, yellow, native), family income (in number of current minimum wage), employment status (active or inactive), non-employment status (unemployed or retired/receiving sick pay), with whom they live (alone, spouse/partner/relatives or others – family members, employees/caregivers), with how many people they live (number of people), place of follow-up (public or private outpatient clinic), presence of comorbidities (Hypertension, Diabetes Mellitus, Dyslipidemia, and/or Atrial Fibrillation), medications on use per day (number), type of medications used besides NOAC (antihypertensive, antidiabetic, antiarrhythmic, and/or lipid-lowering), NOAC in use (dabigatran, apixaban or rivaroxaban), time of use of NOAC (in months), help to take medication (yes or no), frequency of help (number of times a day), interruption of treatment with NOAC in the past month (yes or no), reason for interruption of treatment with NOAC in the past month (surgical or dental procedure, others), adverse events related to the use of NOAC (yes or no), receiving information about NOAC side effects (yes or no), and importance of adherence to NOAC (yes or no).

The MAT was adapted for use in a population on OAC^(14)^. This scale consists of seven items that assess the frequency of forgetting to take medication, carelessness with the time of taking the medication, and not taking the medication related to improvement or worsening of health status, increased dose intake/number of pills associated with the patient’s forgetfulness, interruption of drug treatment associated with running out of the drug, and the frequency of not taking the drugs for reasons that are not indicated by the doctor^(4,14)^. Each item has a six-point ordinal response scale, which varies between 1 (always) and 6 (never). To calculate the final score, the values obtained from the responses to the seven items are added and divided by the number of items, that is, they range from 1 to 6: the higher the score, the better the adherence to the medication. Therefore, the total scores 6 and 5 are counted as value one, resulting in adherence, while the other scores (<5) are recorded as zero, indicating non-adherence, converting it into a dichotomous scale^(4,14)^. In the present study, the analysis of the internal consistency of MAT showed evidence of reliability by presenting a Cronbach’s alpha coefficient of 0.62, classified as acceptable/satisfactory^(15)^.

### Data Analysis and Treatment

Data were entered in an electronic spreadsheet in the software Excel^®^, with double checking of typing and, later, analyzed by the software SAS statistical version 9.4.

Descriptive statistics were used, with the measurement of absolute and relative frequencies for categorical variables, and means, standard deviation, and median for continuous variables. To check data normality, Shapiro-Wilk test was used. For the analysis of comparison between a qualitative variable and two categories in relation to the MAT score, the non-parametric Mann-Whitney test was applied. For the analysis of correlations between the quantitative variables and the MAT scores, Spearman’s correlation coefficient was used, with the following classification for the analysis of the magnitude of the correlation: 0.1 to 0.29 (weak); 0.30 to 0.49 (moderate); and greater than or equal to 0.50 (strong)^(12)^. Multiple linear regression models, via generalized linear models, were built considering the MAT score as a dependent variable. In these models, the estimates of the regression coefficients, as well as their confidence intervals and p-values, were presented. For the nominal variables, the following categories were considered as reference: sex (male), employment status (inactive), help to take medication (no), rivaroxaban as NOAC in use, adverse events (no), interruption of treatment in the past month (no), and private clinic as follow-up location. The significance level adopted for all tests was 5%.

### Ethical Aspects

The study was approved by the Research Ethics Committee (opinion no. 3.985.869) in 2020 and followed all the recommendations of Resolution 466/2012 of the National Health Council. As data collection was performed through telephone call, the free informed consent was recorded and a copy of the Free and Informed Consent Form was sent to the participants through e-mail or digital platform, according to the participant’s preference.

## RESULTS

The study included 120 participants using NOAC, with 72 patients being followed up in a private cardiac clinic and 48 in a public clinic. Just over half of the sample consisted of women (55.8%), with a mean age of 70.1 (standard deviation – SD = 13.8) years, with 9.8 (SD = 6.5) years of study, who lived with 1.7 (SD = 1.5) people on average, with a mean family income of 6.7 (SD = 4.7) minimum wages, representing 59.5% of incomes above two minimum wages. Participants took an average of 5.3 drugs/day (SD = 2.5), including NOACs. Most of them (76.6%) used antihypertensive drugs, followed by the use of antidiabetic (27.5%), antiarrhythmic (44.1%), and lipid-lowering drugs (43.3%). In the sample studied, 33.3% reported the need for help to take medication, with an average frequency of 2.7 times/day (SD = 1.6).

Regarding the NOAC in use, 51.6% of the patients used apixaban or dabigatran and 48.4% used rivaroxaban. The mean time of use of NOAC was 32.3 months (SD = 31.2). Among the participants, 13.3% reported having interrupted treatment with NOAC in the past month. More than half (62.5%) abandoned treatment for reasons other than those related to the surgical or dental procedure, such as lack of financial resources to buy the drug, and, of this total, 60% attended the public clinic. Of the total number of participants, 15% reported an adverse event (thromboembolic or hemorrhagic) related to the use of NOAC. Half of the participants reported having received some information about the side effects of NOAC and 84.1% about the importance of maintaining adherence to this medication. As for the MAT score, the participants were compliant to treatment, with a mean MAT score of 5.7 (SD = 0.3), in a possible range from 1 to 6 (Table 1).

**Table 1.** Sociodemographic and clinical characterization of patients with cardiovascular diseases using new oral anticoagulants (NOAC) (n = 120) – Campinas, SP, Brazil, 2021.

Variables	N	%	Mean (SD)	Median	Range
**Age (years)**			70.1 (13.8)	72.5	25–93
**Sex**					
Female	67	55.8			
Male	53	44.2			
**Color**					
White	90	75.0			
Nonwhite	30	25.0			
**With whom the person lives**					
Family members	101	84.8			
Alone	16	13.4			
Others	02	1.80			
**With how many people the person lives**			1.7 (1.5)	1.0	0–10
**Schooling (years)**			9.8 (6.5)	7.0	0–25
Illiterate	16	13.3			
Some elementary school	55	45.8			
Complete elementary school	05	4.1			
Some high school	00	0.0			
Complete high school	11	9.1			
Some higher education	06	5.0			
Complete higher education	27	22.5			
**Family income (number of minimum wages)**			6.7 (4.7)	5.0	1–30
**Employment status**					
Inactive	106	88.3			
Active	14	11.6			
**Nonemployment status**					
Retired/Sick pay	91	86.6			
Unemployed	14	13.3			
**Adverse events**					
No	102	85.0			
Yes	18	15.0			
**Comorbidities**					
Atrial fribrillation	115	95.8			
Arterial hypertension	84	70.0			
Dyslipidemia	57	47.5			
Diabetes mellitus	36	30.0			
**Follow-up location**					
Private outpatient clinic	72	60.0			
Public outpatient clinic	48	40.0			
Number of drugs in use, including NOAC*			5.3 (2.5)	5.0	1–30
NOAC usage time* (months)			32.3 (31.2)	24	1–180
**MAT**					
MAT total score**			5.7 (0.3)	5.7	4–6
Adherence	117	97.5			
Non-adherence	03	2.5			

*NOAC: new oral anticoagulant; **MAT: Measurement of adherence to treatment.

The analyses of comparison between the scores obtained by the MAT and the sociodemographic and qualitative clinical variables showed that the professionally inactive patients, who used NOAC, were significantly more adherent than the active ones (5.4 *vs* 5.7; p = 0.049). The other variables studied did not show statistically significant relationships (Table 2).

**Table 2.** Comparison of sociodemographic and clinical variables with the Measure of Adherence to Treatment (MAT) scores in patients with cardiovascular diseases using new oral anticoagulants (NOAC) – Campinas, SP, Brazil, 2021.

Variables	n (%)	Mean (SD)	Median	Range	p-value^*^
**Sex**					0.102
Male	53 (44.1%)	5.6 (0.4)	5.7	4.0–6.0	
Female	67 (55.9%)	5.7 (0.3)	5.9	4.7–6.0	
**Employment status**					**0.049**
Active	14 (11.7%)	5.4 (0.5)	5.7	4.0–6.0	
Inactive	106 (88.3%)	5.7 (0.3)	5.7	4.5–6.0	
**Aid to take medication**					0.139
Yes	40 (33.3%)	5.7 (0.3)	5.8	5.2–6.0	
No	80 (66.7%)	5.7 (0.3)	5.7	4.0–6.0	
**Treatment interruption**					0.837
Yes	16 (13.3%)	5.6 (0.5)	5.8	4.0–6.0	
No	104 (86.7%)	5.7 (0.3)	5.7	4.6–6.0	
**Adverse events**					0.798
Yes	18 (15.09%)	5.7 (0.3)	5.7	5.4–6.0	
No	102 (85.0%)	5.7 (0.3)	5.7	4.0–6.0	
**NOAC in use**					0.673
Dabigatran	25 (20.9%)	5.7 (0.3)	5.7	5.2–6.0	
Apixaban	37 (30.8%)	5.7 (0.3)	5.7	4.5–6.0	
Rivaroxabana	58 (48.3%)	5.7 (0.3)	5.7	4.0–6.0	

*p-value obtained using the Mann-Whitney test.

In the analysis of the relationship between the numerical variables and the MAT scores, only the variable time using NOAC showed a significant, negative and weak correlation (p = 0.006), indicating that the longer the NOAC has been used, the lower is adherence to the use of these medications (Table 3).

**Table 3.** Comparison of sociodemographic and clinical variables with the Measure of Adherence to Treatment (MAT) scores in cardiovascular diseases patients on new oral anticoagulants (NOAC) – Campinas, SP, Brazil, 2021.

Variables	MAT r*, (p-value**†**)
**Age**	0.16 (0.078)
**Length of schooling**	−0.05 (0.575)
**Family income**	−0.047 (0.614)
**Number of medicines/day**	0.15 (0.100)
**NOAC usage duration**	−0.25 **(0.006)**

*r = Spearman’s Correlation Coefficient; ^†^p-value = Level of significance.

Finally, in the regression analysis between the set of independent variables and adherence, a positive and statistically significant relationship was observed between family income and inactive employment status and adherence to NOAC. For each increase of one minimum wage in family income, there is an average increase of 0.02 points in the MAT score. In addition, a professionally inactive participant has an average increase of 0.26 points in the MAT score, compared to an active participant.

A statistically significant negative relationship between sex and the use of NOAC was observed, showing that men using NOAC had an average decrease of 0.11 points in the MAT score, compared to women under treatment with NOAC. It was also found that the user of a private outpatient clinic presented an average reduction of 0.13 points in the MAT score, when compared to patients in the public clinic (Table 4). The other independent variables included in the model did not show a statistically significant relationship with the measurement of adherence.

**Table 4.** Results of the multiple linear regression model between sociodemographic and clinical variables (independent variables) and the Measure of Adherence to Treatment (MAT) scores (dependent variable) of patients using new oral anticoagulants (NOAC) – Campinas, SP, Brazil, 2021.

Independent variables	Coefficient	CI* 95%		p-value^**§**^
		LL^†^	UL^‡^	
Intercept	4.98	4.49	5.48	**<0.0001**
Age	0.00	0.00	0.01	0.097
Length of schooling	0.01	−0.01	0.02	0.269
Number of medicines/day	0.02	−0.01	0.04	0.219
Family income	0.02	0.01	0.03	**0.019**
NOAC usage duration	0.00	0.00	0.00	0.109
Sex (Female)	−0.11	−0.23	−0.01	**0.048**
Employment status (Active)	0.26	0.05	0.47	**0.014**
Aid for taking medication (Yes)	−0.02	−0.15	0.12	0.803
NOAC in use (Dabigatran and Apixaban)	0.04	−0.08	0.16	0.517
Adverse events (Yes)	−0.01	−0.17	0.14	0.879
Interruption treatment past month (Yes)	0.13	−0.04	0.29	0.126
Follow-up location (Public outpatient clinic)	−0.13	−0.25	−0.02	**0.025**

*CI: Confidence interval; ^†^LL: lower limit. ^‡^UL: Upper limit; p-value = ^§^Significance level.

## DISCUSSION

This study characterized CVD patients adherence to NOACs, observing the relationship between sociodemographic and clinical variables and adherence to these anticoagulants. The results, in general, corroborate the findings of previous studies that explored this construct in CVD patients using NOAC^(8–10,16)^.

The literature emphasizes that medication adherence is influenced by several factors, such as comorbidities, age, costs, and type of medication in use, frequency, number of medications and complexity of treatment, treatment time, health literacy, as well as beliefs and satisfaction with drug treatment^(2,17)^.

In this study, the patients were classified as adherent to NOAC, considering the high mean MAT score of 5.7. A similar result was found in national and international studies that used MAT to assess medication adherence, but in patients undergoing treatment with OAC^(14,18–19)^ and in patients using antidiabetics^(5)^, in whom the average adherence score was also high, ranging between 4.6 and 6.0. Although these are clinical conditions and medications that are different from those investigated in this study, the comparison is warranted in view of the fact that these are chronic conditions that require the continuous and frequent health monitoring, in which the assessment of adherence also represents a challenge for health professionals^(5,14,18–19)^.

It is observed that MAT showed evidence of reliability in the studied sample, with acceptable internal consistency, evidenced by a Cronbach’s alpha coefficient of 0.62. This finding is similar to that of a Brazilian study that analyzed the properties of MAT measurement in patients using warfarin, whose Cronbach’s alpha coefficient was 0.60^(11)^. In addition, it is consistent with findings from other international and national studies developed with samples using different drugs, whose Cronbach’s alpha coefficient ranged between 0.68 and 0.84^(5,20)^.

As for the clinical profile, among the total number of patients studied, a high prevalence of participants diagnosed with AF (95.8%) was observed. In studies on adherence to NOAC, the predominant profile of patients with AF is represented by males, which differs from the present study, in which 55.8% of the sample were women, but our findings regarding the prevalence in white (75%), hypertensive (70%) people, aged over 60 years (average of 70.1 years)^(8–9,21)^ were corroborated.

With regard to the variable NOAC usage duration, an inverse and statistically significant correlation, although of weak magnitude, was observed between adherence and NOAC usage duration, showing that the shorter the NOAC usage duration, the greater the adherence.

Real-life studies, that is, phase 4 clinical trials with a longitudinal design, in which the use of the drug is outside a controlled research environment, show that patients taking NOAC therapy have high rates of adherence; however, this indicator is gradually decreased after approximately one year of treatment. Such studies, when evaluating the reasons for non-adherence, were faced with the issue of the high cost of NOAC, which over a prolonged period of use even compromises the monthly budget^(8,21)^. Among other factors that can contribute for this finding, the lesser need for outpatient follow-up to monitor the International Normalized Ration (INR) indices and to adjust the doses of patients using NOAC is highlighted. Therefore, there is less interaction between patients and health professionals, reducing follow-up time and moments of instructions about the importance of maintaining adherence to drug treatment^(8)^.

The higher incidence of gastric complaints, such as dyspepsia, specifically in patients using dabigatran, is also considered to be one of the factors negatively interfering with the adherence to NOAC, and this adverse event is the most reported in clinical studies to evaluate the efficacy and safety of these drugs^(21)^. Although a frequent routine of monitoring of anticoagulation levels is not necessary for the treatment with NOAC, the need for periodic and regular control of the liver and kidney functions is highlighted, which may not bother at the beginning of the treatment, but, in the long term, is a factor that shall be considered when managing the treatment^(22)^.

It was also found that there was a prevalence of professionally inactive participants (retired or receiving sick pay), a finding that can be explained by the sample mean age, of 70.1 years. As for family income, the mean was 6.7 minimum wages, represented by 3.9 minimum wages per family member, higher than the national average income, which corresponds to two minimum wages^(23)^. This datum can be explained by the fact that it is related to patients who are mostly from the private outpatient clinic and who, therefore, had socioeconomic conditions to undergo the treatment through health plans/insurance or in a private service. Inactive employment status and income showed a positive and statistically significant relationship with adherence to NOACs, as identified in the literature^(18)^.

International studies show that the relationship between medication adherence and income is directly linked to the onerous cost of treatment with NOAC. Studies carried out in China showed a rate of 6.9% of NOAC discontinuity, explained by the high cost of the drug and the lack of coverage of this treatment by the country’s health insurance^(8,21)^. Therefore, many people cannot afford it, which leads to non-adherence^(21)^.

In the present study, 13.3% of the participants reported having discontinued the use of NOAC in the past month and 62.5% discontinued it for reasons other than those related to a surgical or dental procedure, with one of the main reasons being the high cost of the NOAC treatment. Nationally, unlike what happens with warfarin treatment, NOACs are not offered by the Brazilian Public Health System (SUS)^(24)^.

Economic evaluation is one of the factors analyzed for the decision to incorporate a treatment to SUS. According to the 2016 report of the National Commission for the Incorporation of Technologies – CONITEC, of the Ministry of Health, the value for using NOAC is much higher than that of warfarin, even considering the additional cost of carrying out the INR, which is pointed out as a reason for not incorporating it as a SUS treatment^(24)^. Thus, although with proven clinical efficacy and markedly reduced adverse effects compared to other OACs, NOACs are not accessible to a large portion of the Brazilian low-income population, amd interferes with the adherence of those who, despite having some access, have difficulty maintaining long-term treatment. There is, therefore, the need to develop strategies and public policies that allow reducing the costs of NOAC and its availability at SUS^(24)^.

When analyzing the relationship between the variable sex and medication adherence, inconsistencies are observed in the literature, as there are studies that point to inconclusive results^(25)^, research that reported the prevalence of low adherence in men^(26)^ and others which found female sex as a predictor of non-adherence^(11)^. Therefore, the divergences among the results reflect the difficulty in analyzing the variable sex as an isolated factor, and should therefore be considered together with the socioeconomic and cultural context^(27)^.

In the present study, users of the public outpatient clinic showed better rates of adherence to NOAC, compared to those using private outpatient clinics. It should be noted that some of the participants of the public outpatient clinic participated in clinical research; therefore, they received NOAC they were taking, free of charge, either during or even at the end of the clinical study, a right guaranteed by law^(28)^. In addition, patients, more routinely, received closer support from the research center staff, which may have favored receiving guidance and, therefore, adherence, as observed in a previous study^(29)^.

The patients used, on average, 5.3 types of medication a day, a common finding in other studies and which, despite being consistent with the literature^(9–10,14)^, was not statistically significant in this study. Among these drugs, 48.4% of the patients used rivaroxaban and 51.6% used dabigatran or apixaban. International surveys show that the rates of adherence to rivaroxaban are higher when compared to dabigatran and apixaban, and the explanation for this is related to the frequency of intake: while rivaroxaban is prescribed to be taken once a day, dabigatran and apixaban are prescribed for use two times a day. Thus, the higher the frequency of taking, the lower the adherence^(8,10,21)^.

Moreover, patients who take multiple drugs are more likely to not adhere, which is linked to misconceptions regarding the quantity, frequency, and timing of the medication. When evaluating the prevalence of this relationship in the elderly population, the chances of non-adherence increase dramatically^(9–10)^.

Therefore, strategies to ensure medication adherence, such as monitoring and educational actions by health professionals, help from other people to take the medication, the use of mobile digital technologies, sound alerts, among others, shall be applied^(30)^. In the sample studied, 33.3% reported needing help from other people to take the medication at least once a day, which may have contributed to the high number of adherent participants^(10,14)^.

As limitations for this study, first, there is the use of a self-reported measure of adherence, which has the disadvantage of social desirability, which may have overestimated the measure of adherence. Second, the lack of measurement, in research fields, of laboratory indicators considered the gold standard for direct measurement of adherence to NOACs, such as the Ecarin clotting time test and the Hemoclot^®^ thrombin inhibitor test for dabigatran and the calibrated Anti-Xa in the case of rivaroxaban and apixaban. Third, as this is a cross-sectional study, it was not possible to check cause-and-effect relationships. Fourth, there is the fact that the sample of patients from the public clinic consists in part of clinical research participants, what differentiated them from the other patients in the sample, as they are more exposed to the assistance of the research center staff, and may have contributed to receiving information about adherence. Finally, data collection through telephone call may have interfered in the approach to patients, as some were afraid to answer questions, especially those of a sociodemographic nature, to the researcher, with whom they did not have a bond of care or monitoring of their health treatment, despite all the clarification prior to the interview.

In the current scenario of population aging and chronic diseases, studies to assess global adherence, that is, of drug and non-drug treatment, are highly required. Furthermore, studies are required that broaden the understanding of the use of NOACs by CVD patients and the long-term impact that adherence to this treatment may have in people’s lives, such as safety and satisfaction with the treatment, reduction of complications, and improvement in quality of life.

## CONCLUSION

The results of the present study showed that CVD patients on NOACs showed good adherence to treatment, as measured by the MAT. The factors inactive employment status, higher family income, female sex, and follow-up at a public outpatient clinic were associated with better adherence to NOACs among the participants in this study. These findings contribute to an evidence-based practice, providing a structure for planning preventive interventions related to non-adherence, mainly aimed at CVD patients on NOAC.

## Financial support

This study was financially supported by Fundação de Desenvolvimento da Unicamp – Fundo de Apoio ao Ensino, Pesquisa e Extensão (FAEPEX), No.: 2243/20.
